# Surgical Treatment of a Giant Thoracic Lipoblastoma in a One-Year-Old: A Case Report

**DOI:** 10.7759/cureus.79629

**Published:** 2025-02-25

**Authors:** Angelina Hilendarova, Nikola Kartulev, Zdravka Antonova, Velichka Oparanova, Hristo Shivachev

**Affiliations:** 1 Pediatric Surgery Department, University Multiprofile Hospital for Active Treatment and Emergency Medicine (UMHATEM) "N.I. Pirogov", Sofia, BGR

**Keywords:** childhood, lipoblastoma, thoracic, thoracotomy, tumor

## Abstract

Lipoblastoma (LB) is a rare benign tumor of adipose tissue that typically occurs in infancy and early childhood. Its thoracic location with an intrathoracic extension is exceedingly uncommon.

This study aims to describe a rare case of thoracic lipoblastoma. The main challenges in this case stem from its unusual location and the tumor's considerable size, which elevate surgical and anesthesiology risks.

We present the case of a one-year-old boy with a history of cough and fever, which were treated symptomatically. Incidentally, a chest X-ray revealed a tumor. Computed tomography revealed a giant tumor occupying the entire left pleural cavity. A percutaneous biopsy was performed for verification. Subsequently, a left posterolateral thoracotomy was conducted, and the tumor was completely excised. The procedure carried heightened anesthesiology risks due to compression of the mediastinal organs. Histopathology revealed that the tumor is a benign lipoblastoma, obviating the need for postoperative treatment. The patient recovered uneventfully after the surgery.

Thoracic lipoblastoma in the pediatric population is an extremely rare event, presenting diagnostic and therapeutic challenges. Nonetheless, it should be considered in the differential diagnosis of thoracic tumors in children.

## Introduction

Lipoblastoma (LB) is a rare benign tumor of adipose tissue that typically occurs in infancy and early childhood. Most cases (90%) manifest before the age of three. Males are more commonly affected, with a male-to-female ratio described in many papers ranging from 1.5 to 3:1 [1]. Two forms are described in the literature: a well-circumscribed tumor called lipoblastoma and a diffuse infiltrative form called lipoblastomatosis. While the most typical locations are the extremities and torso, LB can also occur in the head and neck region, mediastinum, and retroperitoneum. Lipoblastoma is usually asymptomatic but may grow rapidly and compress adjacent structures [2].

We present a case of a giant intrathoracic lipoblastoma in a one-year-old boy. This location is extremely rare, with only 20 cases described in the English literature [3]. The main challenges in this case arise from its unusual location and the tumor's considerable size, which increased the surgical and anesthesiology risk.

Lipoblastoma is a benign tumor with a good prognosis after radical excision. It grows rapidly but does not metastasize locally or distally, nor does it undergo malignant transformation [1,2]. Surgery is the treatment of choice. The recurrence rate is 14%-25%, necessitating long-term follow-up [4]. Although rare, lipoblastoma should be considered in the differential diagnosis of thoracic tumors in children [5].

## Case presentation

We present a one-year-old boy with no previous health problems and no significant past medical or family history. He had a normal neurological development and weighed approximately 9 kg. The patient presented with a cough and fever (38.8°C) and was treated symptomatically for four days. Laboratory tests were within normal ranges (leukocytes: 8.5 × 10^9^/L, hemoglobin: 119 g/L, thrombocytes: 386 × 10^9^/L, C-reactive protein (CRP): 0.43 mg/L) (Table 1). On the ninth day after the onset of symptoms, a chest X-ray revealed massive opacification in the left hemithorax with contralateral mediastinal shift (not available, performed in a different medical facility). The child was then admitted to the Department of Pediatric Thoracic Surgery for further examinations. On examination, the child presented with tachypnea, exhibiting a respiratory rate of 35-40 breaths per minute and a SpO2 of 94% on room air. His skin appeared pale, and he had a fever of 37.7°C. Auscultation revealed normal breath sounds over the right lung field, while they were absent on the left. There was no observable movement in the left hemithorax during respiration. The abdominal examination was unremarkable, with normal peristalsis and no tenderness to palpation. Blood and biochemical tests were within the normal range, with slightly increased inflammation markers (leukocytes: 14.94 × 10^9^/L, hemoglobin: 119 g/L, hematocrit: 0.37 L/L, thrombocytes: 370 × 10^9^/L, CRP: 3.33 mg/dL, aspartate transaminase (AST): 41 U/L, alanine aminotransferase (ALT): 18 U/L, creatinine: 41 µmol/L) (Table 2).

**Table 1:** Patient's laboratory test on the day of the onset of symptoms CRP: C-reactive protein

Laboratory test	Patient's result	Normal range
Leukocytes	8.5 × 10^9^/L	5.1-13.0 × 10^9^/L
Hemoglobin	119 g/L	108-138 g/L
Thrombocytes	386 × 10^9^/L	180-440 × 10^9^/L
CRP	0.43 mg/L	0.00-0.50 mg/L

**Table 2:** Patient's laboratory tests on the day of admission to the Department of Pediatric Thoracic Surgery CRP: C-reactive protein, AST: aspartate transaminase, ALT: alanine aminotransferase

Laboratory test	Patient's result	Normal range
Leukocytes	14.94 × 10^9^/L	5.1-13.0 × 10^9^/L
Hemoglobin	119 g/L	108-138 g/L
Hematocrit	0.37 L/L	0.32-0.41 L/L
Thrombocytes	370 × 10^9^/L	180-440 × 10^9^/L
CRP	3.33 mg/dL	0.00-0.50 mg/L
AST	41 U/L	15-60 U/L
ALT	18 U/L	13-45 U/L
Creatinine	41 µmol/L	45-105 µmol/L

**Figure 1:**
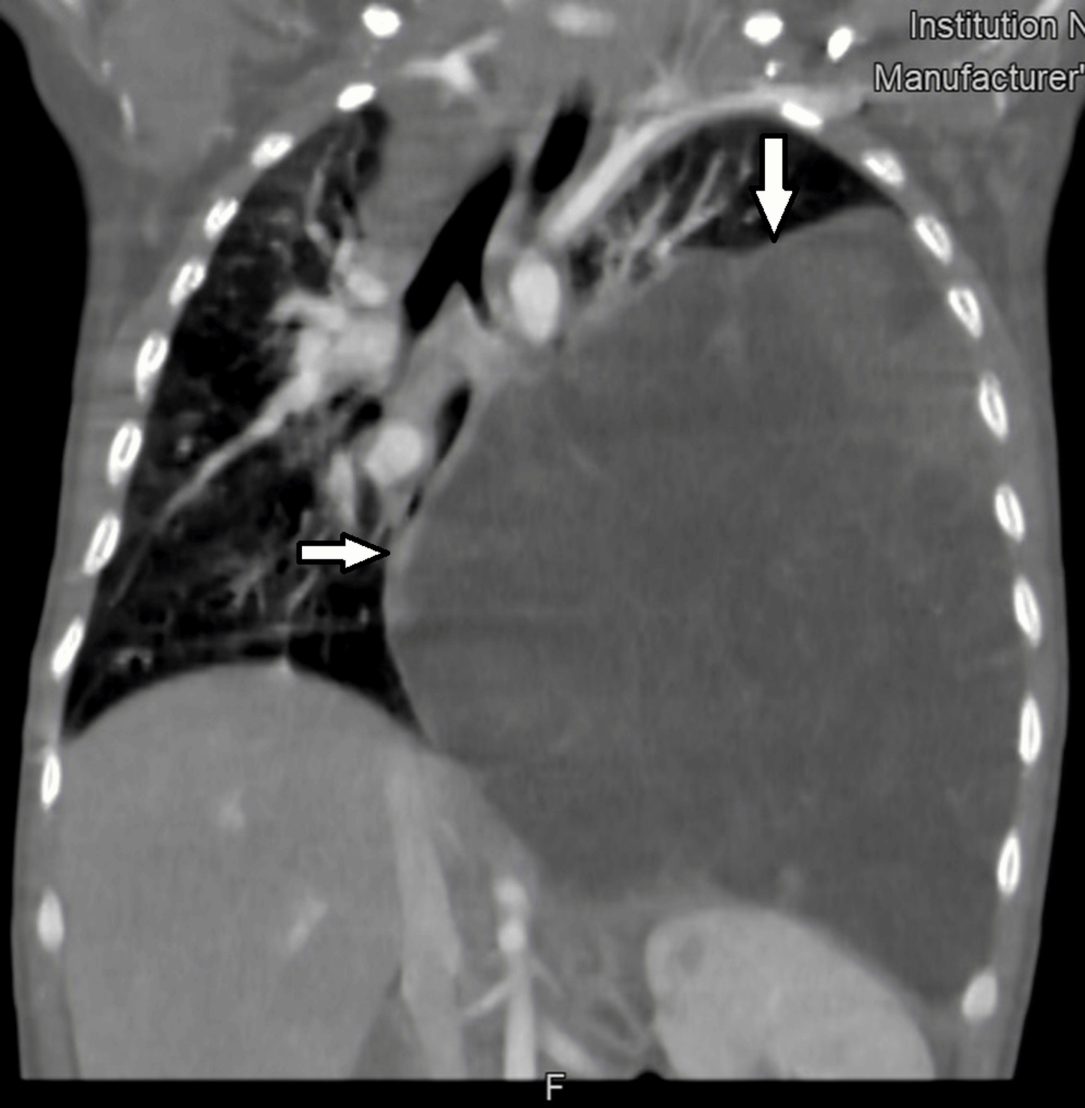
CT scan (coronal view): a large mass with predominantly fat density is seen in the left thoracic cavity, causing mediastinal shift to the right with esophageal, tracheal, and thoracic aorta displacement CT: computed tomography

**Figure 2:**
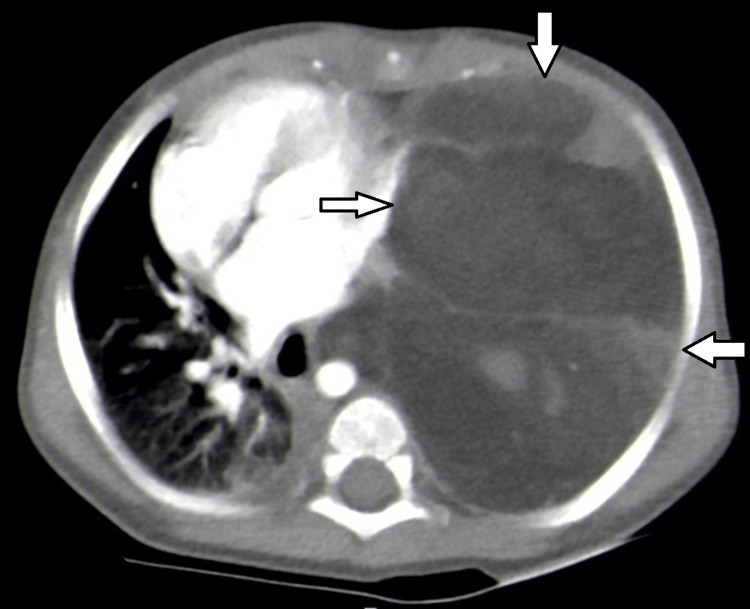
CT scan (axial view): a fat density mass with intratumoral stranding in the left hemithorax showing significant cardiac displacement to the right CT: computed tomography

Tumor markers were negative (beta-human chorionic gonadotropin and alpha-fetoprotein). Contrast-enhanced computed tomography (CT) revealed a giant tumor with predominantly fatty density, irregular septations, intratumoral stranding, and soft tissue nodular parts internally. The tumor measured 96 × 98 × 89 mm. Cardiac, esophageal, tracheal, and thoracic aorta displacement was observed (Figure 1 and Figure 2). The left main bronchus exhibited a reduced diameter, and the left lung was atelectatic. Mediastinal lymph nodes were not enlarged. Thymus hyperplasia was also found.

Cardiology consultation revealed dextroposition of the heart without any functional or structural abnormalities. Oncohematology consultation suggested that the tumor was a teratoma. They recommended biopsy and surgery, with chemotherapy considered only after histopathological evaluation. An ultrasound-guided percutaneous biopsy was performed, and histopathology showed only the presence of adipose tissue in the sample.

**Figure 3:**
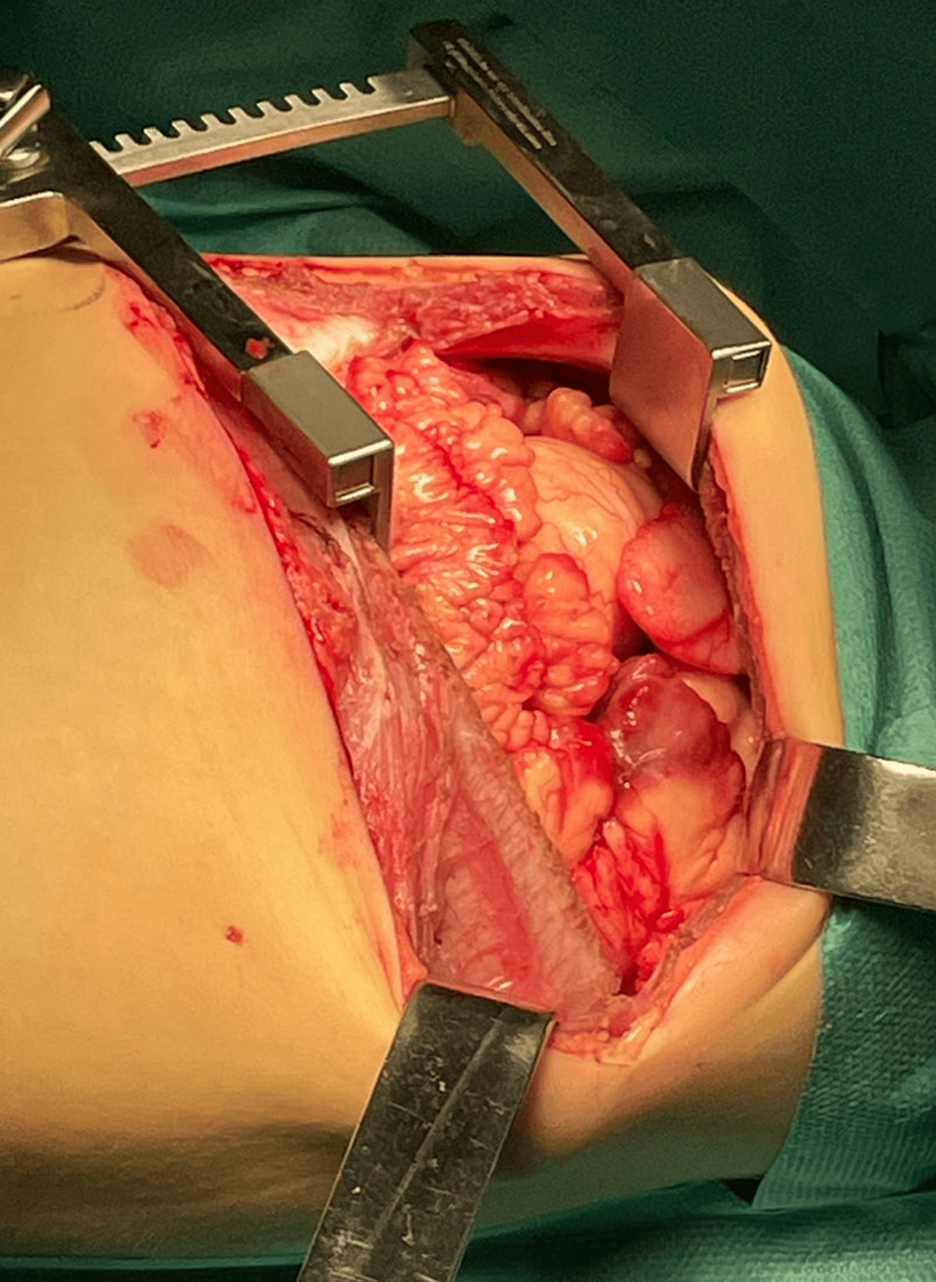
Left posterolateral thoracotomy: a giant lobulated yellowish tumor, occupying almost the entire left hemithorax, is presented

**Figure 4:**
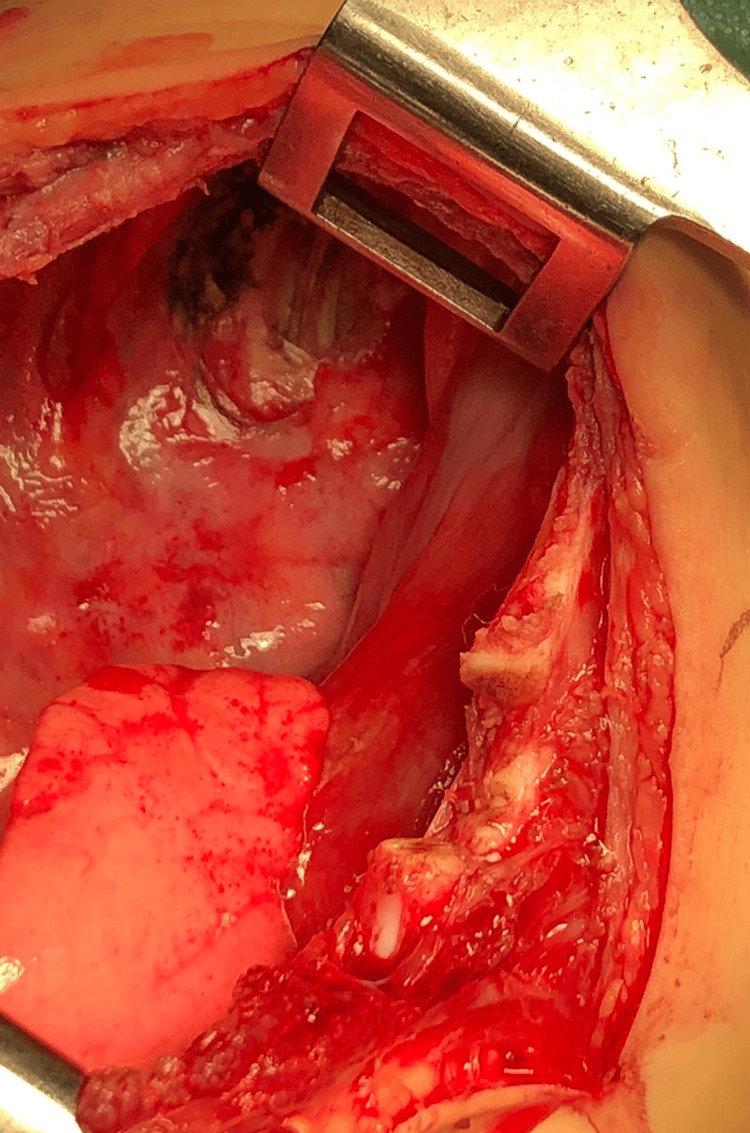
Intraoperative view after the radical tumor extirpation

**Figure 5:**
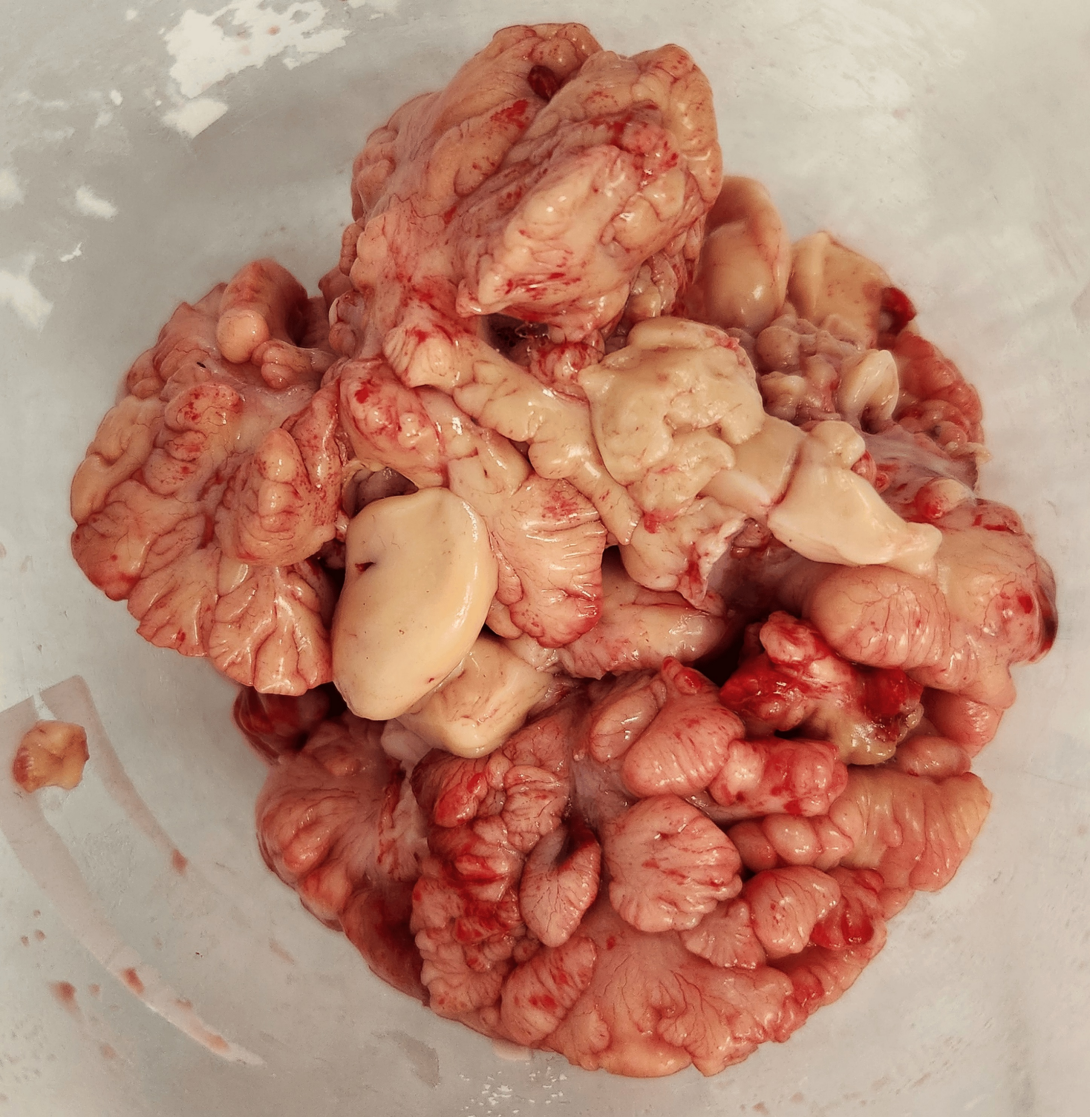
Radical extirpated tumor sent for histopathological examination

After a multidisciplinary consultation involving a pediatric surgeon, an anesthesiologist, a pediatrician, and a hematologist-oncologist, a decision for surgery was made. The procedure carried heightened surgical and anesthesiology risks due to the compression of the mediastinal organs. A left posterolateral thoracotomy was performed in the fifth intercostal space under general anesthesia with single-lumen endotracheal intubation. A giant lobulated yellowish tumor was found, occupying almost the entire left hemithorax (Figure 3). The tumor was attached to the parietal pleura with a pedicle in the costodiaphragmatic angle at the level of the 10th rib. Radical extirpation was performed with blunt and electrocautery dissection after ligation and division of the pedicle, and the tumor was sent for histopathological examination (Figure 4 and Figure 5). A drain tube was inserted into the pleural cavity, and the patient was admitted to the Pediatric Intensive Care Unit.

**Figure 6:**
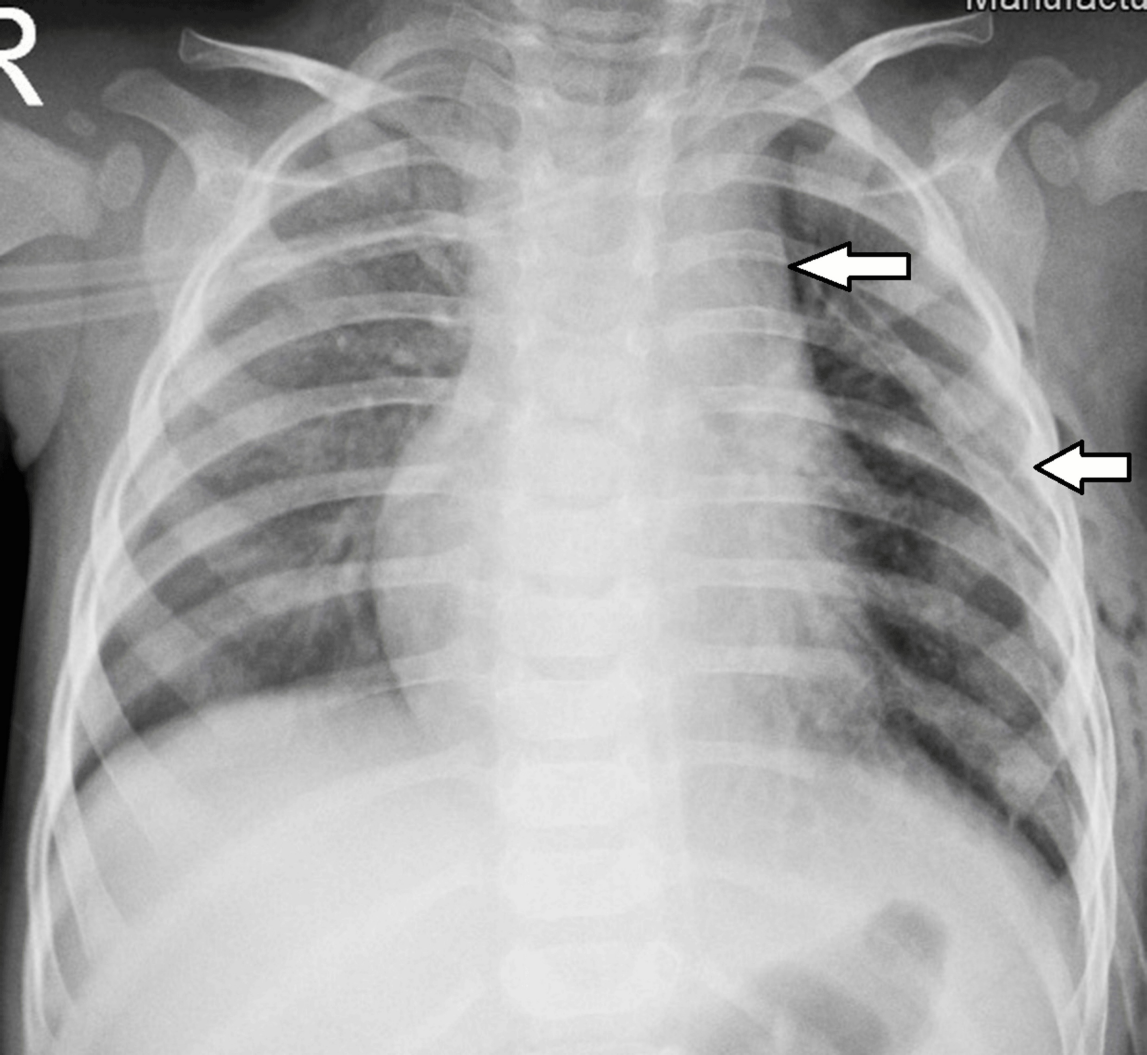
Anterior-posterior chest X-ray on the third postoperative day showing complete re-expansion of the left lung with no mediastinal shift

**Figure 7:**
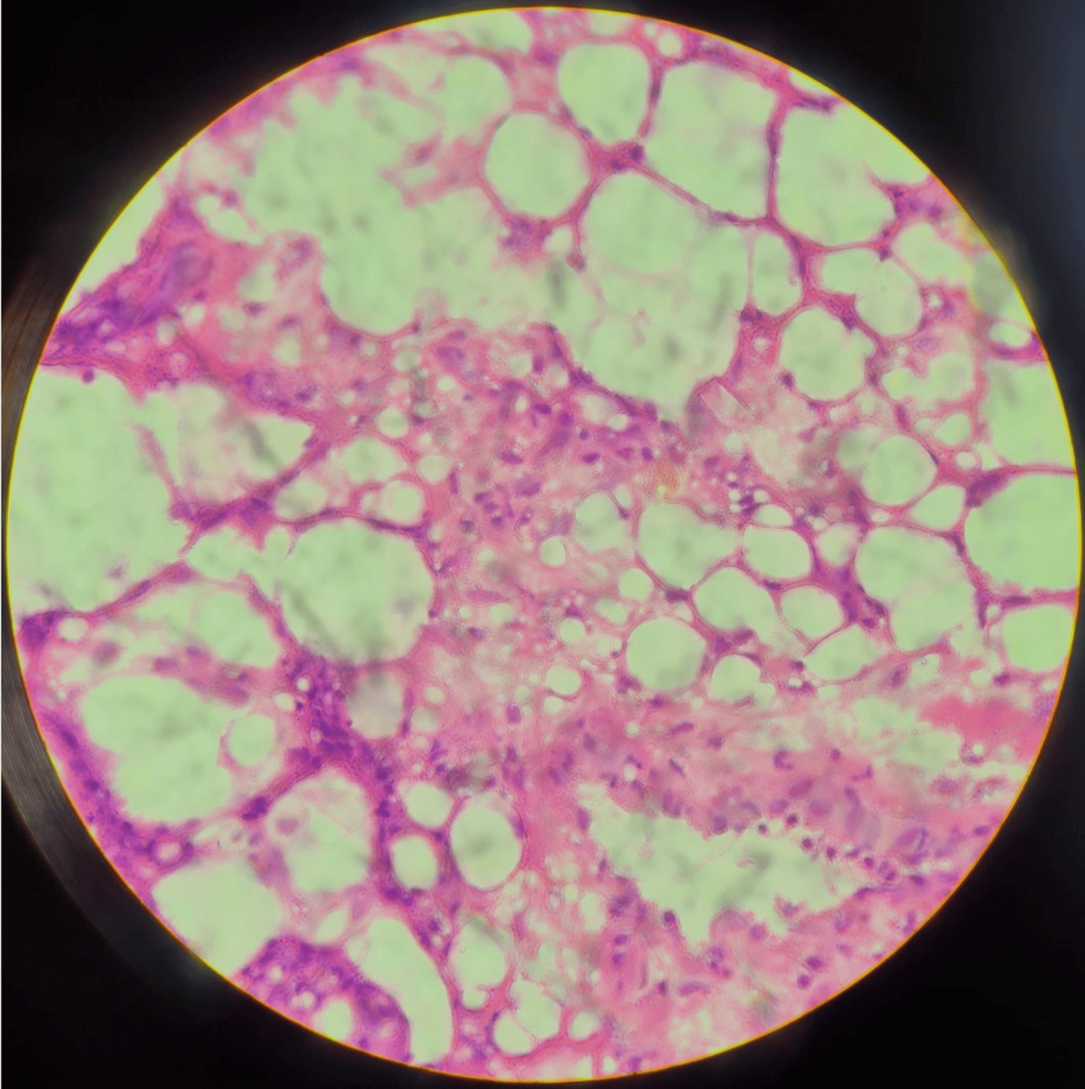
Histopathology: hematoxylin and eosin frozen section (×400)

The postoperative period was uneventful. Postoperative therapy included antibiotics (sultamicillin), analgesia, saline inhalations, and rehabilitation. On the third postoperative day, a chest X-ray was performed and showed complete re-expansion of the left lung with no mediastinal shift (Figure 6). The chest tube was removed on the same day, and he was discharged on the fifth postoperative day. Histopathology revealed a mesenchymal encapsulated lobulated tumor with lipoblast differentiation. Immature primitive mesenchymal cells and lipoblasts were found in the periphery of the tumor, while mature cells were found in the center (Figure 7). The conclusion was that the tumor was a benign lipoblastoma. No further treatment was indicated for our patient. Clinical and ultrasound examinations were conducted at the third, sixth, and 12th months after surgery with no recurrence of the tumor. Further follow-up is planned for the next five years, including clinical examinations and imaging studies. The prognosis is favorable, with a low recurrence rate for lipoblastoma [4,5].

## Discussion

Lipoblastoma is a rare benign mesenchymal tumor of embryonic fat tissue composed of adipocytes and lipoblasts, typically occurring in infancy and early childhood [1,2]. Most cases (90%) occur before the age of three, with 40% described in the first year of life [1,4,6,7]. Phuyal et al. describe that lipoblastoma accounts for only 3% of soft tissue tumors in the first year of life and 1%-2% of all benign tumors in children [2]. Male prevalence is reported in many papers, with a male-to-female ratio of 1.5-3:1 [1,4,6,8,9].

Lipoblastoma was first described by Jaffe in 1926 [9]. In 1958, Vellios et al. described two histopathological types: a well-circumscribed tumor called lipoblastoma and a diffuse form called lipoblastomatosis [1,9,10]. The first type is localized, superficial, and encapsulated (found in about 70% of cases). The second type is multicentric, non-capsulated, and infiltrative (found in about 30% of cases) [1,6]. Lipoblastoma is more common and tends to occur superficially, while lipoblastomatosis occurs in deeper tissues [2].

Lipoblastoma may arise anywhere within the soft tissue where mesenchymal cells are present [4,6]. Baruah et al. determined the pathogenesis of lipoblastoma to be embryonic white fatty cells that continue to proliferate and develop during the postnatal period [4]. Some authors suggest that lipoblastoma could differentiate into a lipoma if followed up long enough [11]. Other studies have described cases of spontaneous regression [3].

The most typical locations for lipoblastoma are the extremities and the trunk, where immature adipose elements persist [7-9,12], but it can also be found in the head and neck region, mediastinum, and retroperitoneum [4,13]. Chest wall lipoblastoma with an intrathoracic extension is very rare, and only a few cases are described in the literature [1,2,4]. Other rare locations include the axilla, parotid gland, eyelid, tonsillar fossa, omentum, mesentery, scrotum, labia, inguinal region, perineum, gluteal region, heart, and lung [8,9,14-16].

Clinically, it is very difficult to distinguish lipoblastoma from a malignant tumor [17]. Lipoblastoma does not occur in association with clinical syndromes or other neoplasms [10]. In most cases, lipoblastoma is asymptomatic, but due to its potential for rapid growth, it may become symptomatic by compressing adjacent organs and structures [6,18,19]. Symptoms depend on the location and size of the tumor [6]. Lipoblastoma of the extremities may present only as a rapidly growing painless swelling. In cases of thoracic, mediastinal, pleural, pulmonary, or lower neck localization, symptoms could include recurrent respiratory infections, cough, wheeze, stridor, dyspnea, and even signs of acute respiratory failure with life-threatening asphyxia [6,7,20,21]. The tumor could cause airway compression, leading to poor airway clearance and increasing predisposition to infections [1]. Vascular compression may result in superior vena cava syndrome. Rare symptoms of spinal cord compression are also described in the literature [22]. Mesenteric, omental, and retroperitoneal lipoblastoma can present with symptoms such as abdominal pain, vomiting, diarrhea, and anorexia [6]. Lipoblastoma may cause bone enlargement in some cases, which can complicate surgery [2,23]. In our patient, the tumor was well-circumscribed, and no muscle or bone involvement was observed.

Preoperative imaging is not always diagnostic but can provide useful information about the extent of the tumor and aid in surgical planning [6]. The first diagnostic tool in children should be ultrasound [24,25]. This is the fastest and safest modality to evaluate any tumor in the pediatric population. Doppler imaging may reveal a hyperechoic and relatively homogeneous tumor [26]. Chest X-ray findings are nonspecific and depend on the tumor size, thickness, and location. Typically, opacification without calcification or bone erosions is observed, and a mediastinal shift may be evident. In rare cases, bone enlargement may be present.

A CT scan typically shows nonspecific heterogeneous low-density fat tissue with some septations and no calcifications [5,27]. It can reveal the composition of the tumor, including the proportion of adipose tissue versus myxoid stroma, and the presence of internal fibrous tissue, vasculature, and soft tissue nodules. Myxoid components are predominant in infants and young children, while findings of fat tissue are typical for older children [6]. However, these findings may not differentiate between benign and malignant lipomatous tumors, meaning that a CT scan alone cannot provide a definitive diagnosis [4,10,28,29]. Nevertheless, it can provide useful information about the origin of the tumor, its size, and its anatomical extent.

Magnetic resonance imaging (MRI) is considered the best diagnostic tool for preoperative diagnosis of lipoblastoma [29]. It typically shows intermediate to high signal intensity on T1-weighted images depending on the amount of immature fat [4,5,19]. Zahrae et al. define MRI as the gold standard for preoperative imaging in lipoblastoma [24]. In our case, we were unable to perform an MRI due to heightened anesthesiology risks. While CT scan and MRI can be very helpful in cases of lipoblastoma, the definitive diagnosis should be made by histopathological examination [26]. Fine needle biopsy may not provide accurate information about the diagnosis [7,27]. Laboratory tests are usually normal with negative tumor markers.

The differential diagnosis includes other fat-containing tumors such as lipoma, liposarcoma, hibernoma, angiolipoma, myolipoma, teratoma, and myxoid tumors [9,15,30]. It is crucial to differentiate between benign and malignant lipomatous tumors because their management and prognosis differ. Liposarcoma is typical in adults and children over 10 years old, and it is extremely rare in children under three years of age [6,17,18]. Teratomas may contain calcifications or ossifications in imaging studies [19]. Myxoid tumors may include hemangioma with invasion of adipose tissue or encasing a portion of it. Other benign tumors such as lipoma and hibernoma present with similar clinical findings to lipoblastoma, but they grow very slowly [17]. Hibernoma is a fatty tumor that arises from fetal brown fat [24]. It is very difficult to distinguish lipoblastoma from other fat-containing tumors preoperatively [27].

Histopathology is the only definitive method for diagnosing lipoblastoma. Microscopically, lipoblastoma is characterized by a proliferation of mature and immature fat cells, along with mesenchymal precursors and lipoblasts in various stages of differentiation, within a myxoid stroma with fibrous septa [31,32]. No nuclear atypia or pleomorphism is observed, and the mitotic rate is extremely low [6]. In immunohistochemistry (IHC), adipocytes typically stain positive for CD34 and S100 protein, while primitive cells are positive for desmin [8]. Lipoblastomas also express leptin and leptin receptors [33]. The main difference between lipoblastoma and lipoma is the presence of lipoblasts. Liposarcoma differs from lipoblastoma in its lack of lobulation, variable growth pattern, and presence of nuclear atypia [6,18]. Lipoblastoma is MDM2- and CDK4-negative, while liposarcoma is positive. Routine IHC is recommended for all pediatric lipomatous tumors, as it helps in accurate diagnosis [32].

Recent cytogenetic studies have described clonal chromosomal aberrations involving rearrangements of chromosome 8q11-q13, with the participation of the pleomorphic adenoma 1 (*PLAG1*) oncogene [29,30,32,33]. This gene encodes a zinc finger transcription factor, and its overexpression is believed to be the primary transforming event in lipoblastoma development [31]. Harrer et al. postulate that the region 8q11.2-q13 is critical for the development of lipoblastoma [11]. According to the literature, approximately 70% of lipoblastomas exhibit rearrangements of the *PLAG1* gene, and up to 18% have polysomy of chromosome 8 [33].

Complete surgical resection is the treatment of choice for lipoblastoma [4,6,9,10,18]. A wide "cancer" type of surgery should be avoided, especially in infants [17]. Radical extirpation is the best therapeutic option to reduce recurrences and provides an excellent prognosis [1,6,19,27]. Lipoblastoma does not metastasize locally or distantly or undergo malignant transformation [1,2,4,10,17,27,34]. Chemotherapy is not necessary in these patients [24]. Regular follow-up is recommended for the next five years, including clinical examinations and imaging studies to monitor for potential recurrences.

The recurrence rate of lipoblastoma is reported to be 14%-25%, necessitating long-term follow-up [7,9,11,16,22,27,35]. It is higher if the tumor is not completely extirpated or in cases of diffuse disease [35]. Lipoblastomatosis has an even higher recurrence rate [15]. In case of recurrence, re-excision is indicated [11,22,35]. Ghallab et al. recommend a minimum follow-up of five years to prevent recurrences [6,7,15,24,35]. Clinical and imaging examinations are essential for follow-up, and some authors suggest cytogenetic analysis for the *PLAG1* gene [9].

## Conclusions

Lipoblastoma is a rare benign tumor of infancy and early childhood, and its intrathoracic localization is extremely uncommon. The rapid growth and significant size of the tumor can pose challenges in both diagnosis and treatment. Despite its rarity, it should be considered in the differential diagnosis of thoracic and mediastinal tumors. Surgical excision is the treatment of choice. We recommend rigorous follow-up with clinical examinations and imaging studies for at least five years after surgery to minimize the risk of recurrence.
